# Creeping Bentgrass Yield Prediction With Machine Learning Models

**DOI:** 10.3389/fpls.2021.749854

**Published:** 2021-11-04

**Authors:** Qiyu Zhou, Douglas J. Soldat

**Affiliations:** Department of Soil Science, University of Wisconsin–Madison, Madison, WI, United States

**Keywords:** turfgrass, nitrogen management, yield prediction, machine learning, random forest

## Abstract

Nitrogen is the most limiting nutrient for turfgrass growth. Instead of pursuing the maximum yield, most turfgrass managers use nitrogen (N) to maintain a sub-maximal growth rate. Few tools or soil tests exist to help managers guide N fertilizer decisions. Turf growth prediction models have the potential to be useful, but the currently existing turf growth prediction model only takes temperature into account, limiting its accuracy. This study developed machine-learning-based turf growth models using the random forest (RF) algorithm to estimate short-term turfgrass clipping yield. To build the RF model, a large set of variables were extracted as predictors including the 7-day weather, traffic intensity, soil moisture content, N fertilization rate, and the normalized difference red edge (NDRE) vegetation index. In this study, the data were collected from two putting greens where the turfgrass received 0 to 1,800 round/week traffic rates, various irrigation rates to maintain the soil moisture content between 9 and 29%, and N fertilization rates of 0 to 17.5 kg ha^–1^ applied biweekly. The RF model agreed with the actual clipping yield collected from the experimental results. The temperature and relative humidity were the most important weather factors. Including NDRE improved the prediction accuracy of the model. The highest coefficient of determination (R^2^) of the RF model was 0.64 for the training dataset and was 0.47 for the testing data set upon the evaluation of the model. This represented a large improvement over the existing growth prediction model (*R*^2^ = 0.01). However, the machine-learning models created were not able to accurately predict the clipping production at other locations. Individual golf courses can create customized growth prediction models using clipping volume to eliminate the deviation caused by temporal and spatial variability. Overall, this study demonstrated the feasibility of creating machine-learning-based yield prediction models that may be able to guide N fertilization decisions on golf course putting greens and presumably other turfgrass areas.

## Introduction

There are 34,011 golf courses in the world, and 45% of them (15,372 golf courses) are in the USA according to a report in 2016. An average course has about 30 hectares of maintained turf, and there are over 1,000,000 hectares of maintained turf on the golf courses in the USA. Golf course turfgrass is usually intensively fertilized, and nitrogen (N) is applied in the greatest quantities out of all nutrients. Gelernter et al. (2016) estimated that US golf courses use 55,333 mg of N annually. These N inputs pose a significant non-point source pollution risk (Bock and Easton, 2020). Hence, there is a need to optimize N management on golf courses.

Nitrogen fertilization is one of the most important management practices that affect many characteristics of golf course putting greens, including density, color, shoot and root growth, wear and temperature tolerance, thatch accumulation, and disease susceptibility (Beard, 1972; Carrow et al., 2002; Frank and Guertal, 2013). Nitrogen is often the most limiting nutrient on putting greens and is, therefore, an important driver of plant growth. Relatively high annual N fertilization rates result in verdant and aesthetically pleasing playing surfaces. However, the rapid growth that results in increasing thatch and soil organic matter content can reduce the function and aesthetics of putting greens (Meinhold et al., 1973; Murray and Juska, 1977; Throssell, 1981; Gaussoin et al., 2013). On the other hand, if the N fertilization is relatively low, it can be hard for putting greens to recover from ball marks and wear damage which encourages weed invasion (algae, moss, annual bluegrass, etc.) (Beard, 1972). Nitrogen management is clearly of great importance to ensure the quality of putting greens, yet there are only a few quantitative methods for determining or estimating the N requirements of putting green turfs. Most turf managers make N application decisions based on the visual appearance of the turfgrass, performance, environmental concerns, and budget (Throssell et al., 2009; Frank and Guertal, 2013), but rarely based on the turfgrass growth rate and N removal from the clipping harvest. Therefore, quantitative methods to assess N fertilization requirements would represent an important advance in the precision of N management of putting greens.

The N cycle of putting greens on sand root zones can be simplified. Potential N loss pathways, including denitrification, volatilization, runoff, and leaching, are typically negligible when the best management practices are followed (Snyder et al., 1984; Morton et al., 1988; Gross et al., 1990; Miltner et al., 1996; Erickson et al., 2001, 2008). This leaves clipping removal as the primary output of N, and N fertilization as the primary input. When the clipping removal exceeds the N input, the soil organic matter will decrease. When the annual N fertilization exceeds the clipping removal, the soil organic matter will increase. This very simple conceptual model highlights the importance of quantifying N removal in clippings. Because the tissue N content of creeping bentgrass is relatively stable, the annual N removal can be approximated by the dry matter removed from mowing (Kussow et al., 2012; Zhou and Soldat, accepted). Therefore, an accurate grass yield production prediction model could be useful for estimating the N removal from putting greens.

Generally, two methods are often used for plant yield prediction: biophysical models and statistical models. Biophysical models predict plant growth by stimulating plant growth, nutrient cycling, as well as water and energy balance on regular time steps. Briefly, a biophysical model simulates plant growth based on physical and physiological processes. The DAYCENT and CENTURY models (Bandaranayake et al., 2003; Qian et al., 2003; Zhang et al., 2013b) are two agroecosystem models that can be used for monitoring turf productivity, soil organic matter changes, and environmental impacts caused by different management practices. Despite their success, two major limitations still exist in the biophysical models: (1) they usually make relatively long-term yield predictions. However, for turfgrass management on a golf course, especially on the greens, a prediction at a finer scale (daily or weekly) is essential to guide precision N fertilizer applications; (2) the model calibration is quite challenging and requires intensive data collection from the field to field, which is less practical to be widely used by turfgrass managers. Although biophysical models often fail to represent short-term turfgrass biomass production, they help provide management decisions by successfully simulating soil organic carbon and N dynamic with various management practices (Bandaranayake et al., 2003; Qian et al., 2003; Chang et al., 2013) and tracing the fluxes of carbon and N gases (Parton et al., 1998; Del Grosso et al., 2006, 2008; Zhang et al., 2013a).

On the other hand, statistical models were developed by establishing empirical relationships between input variables and ground reference data. The most commonly used turfgrass yield prediction model, the PACE Turf growth potential (GP) model was proposed by Gelernter and Stowell (2005). The PACE Turf GP model uses temperature to estimate the relative growth potential of both warm-season and cool-season grasses. The model assumes that 20°C is optimal for cool-season grass growth. When the average daily temperature is 20°C, the growth potential of cool-season turfgrass is at 100%, as the temperature increases or decreases from 20°C, the relative growth potential decreases until it approaches 0% near 0 and 40°C. The disadvantage of the model is that it requires users to make assumptions about the actual growth rate at 100% relative growth. In addition, this model fails to consider the factors that influence turfgrass growth aside from temperature. More complex statistical models can be constructed using more variables to fit historical data on plant yields and weather to build empirical predictive algorithms. The advantage of statistical models over biophysical models is that statistical models require less extensive information on the plant characteristics, management practices, soil, and canopy conditions, and statistical models are easier to calibrate using existing data (Lobell and Burke, 2010).

Various machine learning models have been developed for agricultural crop yield prediction including the linear regression model (Bolton and Friedel, 2013; Ramesh and Vardhan, 2015), support vector regression (Jaikla et al., 2008; Brdar et al., 2011), and decision tree (Veenadhari et al., 2011). These approaches only utilize a single regression model when making predictions, and some machine learning models are only capable of solving linear problems or are likely to occur overfitting when the number of training data is limited (Pal, 2007). Overfitting can cause the model to have high variance or make a poor prediction on the testing data. With the increasing demand for a more accurate yield prediction and guidance for precision N management, studies have tested machine-learning models that build on several base learners to avoid overfitting and increase the prediction accuracy (Zhang and Crawford, 2015). The ensemble methods, such as bagging and boosting, combine the predictions of several models and are capable of solving non-linear problems. These models also largely avoid overfitting and usually have a higher prediction accuracy (Belgiu and Drăguţ, 2016). Recently, ensemble models, such as the random forest (RF) model which is a representative of the bagging ensemble method, have been developed to predict crop yield in response to climate variables (Lobell et al., 2007; Tulbure et al., 2012; Fukuda et al., 2013; Newlands et al., 2014; Zhang et al., 2019), and has been recognized as an important advancement for agricultural industries (Everingham et al., 2016; Chlingaryan et al., 2018; van Klompenburg et al., 2020). Random forest uses a decision tree as a base learner and generates many decision trees in parallel. The gradient boosting model is an example of a boosting ensemble method, which also uses a decision tree as a base learner, and has been used in agricultural crop yield prediction (Charoen-Ung and Mittrapiyanuruk, 2018). Compared with RF, the gradient boosting model builds shallower trees, and these trees are generated based on the mistake of the previous trees. Extreme gradient boosting was introduced recently and has been recognized as an advanced gradient boosting method. Extreme gradient boosting has been a winning tool for several machine learning competitions (Phoboo, 2014) due to its high efficiency and accuracy, and has been tested on agricultural crop yield prediction (Herrero-Huerta et al., 2020). Agricultural and turfgrass production systems have many important differences, and therefore the ability of machine learning techniques to be useful in turfgrass management needs to be tested to determine their potential feasibility.

The goal of this study was to build and evaluate several machine learning models and to predict golf courses putting green creeping bentgrass yield. If successful, such models could become decision support tools for N fertilization in golf course management.

## Materials and Methods

### Study Sites

The clipping yield data used to build the growth model were obtained from a series of research trials conducted at the University of Wisconsin-Madison O.J. Noer Turfgrass Research and Education Facility located in Verona, WI, United States. The field experiments were conducted on two different sand-based putting green root zones from 2019 to 2020, both constructed according to the recommendations of the US Golf Association (USGA) (US Golf Association [USGA], 2004). The root zone characteristics are reported in Table 1. The grass on both greens was “*Focus*” creeping bentgrass (*Agrostis stolonifera*), which is one of the most commonly used cool-season species for golf course putting greens. The research plots were maintained using typical practices of putting green maintenance at golf courses with creeping bentgrass in the northern USA. The plots were mowed five times a week at the height of 3.2 mm, irrigated daily to replace evapotranspiration (ET) as estimated by an on-site weather station (except when irrigation was a treatment), and fertilized with approximately 100 kg N ha^–1^yr^–1^ split into 10 applications of 10 kg N ha^–1^ as urea (except when N fertilizer was a treatment). The research areas were topdressed with approximately 0.6 m^3^ ha^–1^ of sand every 3 weeks during the growing seasons. Hollow tine cultivation was conducted once at the end of each growing season and the holes were filled with topdressing sand. Diseases and other pests were controlled as needed. To examine the feasibility of the machine learning models for predicting the clipping production of creeping bentgrass at locations other than the site where the models were constructed, we evaluated the performance of the models for the creeping bentgrass yield production at a golf course within 20 km of Minneapolis, MN, United States; Minneapolis is approximately 400 km northwest of the research site in Madison, Wisconsin.

**TABLE 1:** Soil chemical properties of two putting green root zones used for creating or evaluating the growth prediction models.

	Depth (cm)	SOM^a^ (%)	P^b^	K	Ca	Mg	CEC^c^ (cmol kg^–1^)	pH
					
			(mg kg^–1^)					
Research green 1	0 – 5	1.23	64.2	91.6	1210	295	8	7.5
	5 – 10	0.55	17.0	25.5	579	144	4	7.3
Research green 2	0 – 5	0.67	25.9	40.7	487	133	3	7.7
	5 – 10	0.51	24.1	17.2	430	102	3	7.5

*^*a*^SOM, soil organic matter by loss on ignition (360°C for 2 h).*

*^*b*^nutrients extracted via Mehlich-3 method (Mehlich, 1984).*

*^*c*^CEC, cation exchange capacity via summation of extracted cations.*

### Management Practices Affecting Yield

To develop an accurate estimation of the turfgrass yield with a machine learning model, it is critical to include the factors that affect the growth rate of creeping bentgrass, thus, data from a series of studies was used. The goal of this section is not to present the results of the individual studies, but rather to utilize the data from these studies for statistical model development. A brief and partial description of the experiments from which the data were obtained follows.

#### Experiment 1. Creeping Bentgrass Growth Response to Soil Moisture Content, N Fertilization, and Traffic

The study was conducted in 2019 on the research greens listed in Table 1 and was designed to explore the combined effects of N fertilizer, walking traffic, and soil moisture content on creeping bentgrass growth. The experimental design was a randomized split-plot (1.2 m by 2.4 m) design with soil moisture level as the main plots (3.6 m by 2.4 m) and the sub-plots received three different traffic intensities. The N fertilizer rates were applied as 0 and 5 kg N ha^–1^ biweekly. The soil moisture contents were maintained at 9–15, 17–22, and 25–29% as measured by time-domain reflectometry with 7.6 cm rods (FieldScout TDR 350, Spectrum Technologies, Aurora, IL, United States). These soil moisture contents were selected to represent low, mid-range, and excessive water content for the sand-based putting green. The soil moisture was measured before each clipping collection event, with three measurements averaged to represent the moisture in each plot. If the soil moisture was below the assigned level, irrigation was then applied by hand watering to meet the requirement. Three traffic intensities (control, medium, and high) were given based on an observational trial conducted by Hathaway and Nikolai (2005) at the Forest Akers West Golf Course on the 13th green around the hole in East Lansing, MI, United States. Then, traffic was applied by five researchers by walking on the plots wearing golf shoes at a speed of 95 steps/min between 1,300 and 1,500 h from Monday through Friday until the treatments reached the assigned weekly traffic intensity requirement. The high traffic intensity plot received around 760 steps/week which represented 1,400 rounds/week, the medium traffic intensity plot received about 380 steps/week that represented 700 rounds/week, and the control plot that did not receive traffic treatment.

#### Experiment 2. Creeping Bentgrass Growth Response to a Wider Range of N Fertilization

The study was conducted in 2019 and 2020 on the two research greens listed in Table 1 to monitor the creeping bentgrass growth when a wide range of N fertilizer was applied. The experiment was also completed with a randomized design with three replications each, and each plot measured 1.2 × 2.4 m. The N fertilization rate ranged from 0 to 17.5 kg N ha^–1^ biweekly using urea as the N source. All the plots received a traffic intensity of 1,000 rounds/week and obtained regular disease control and irrigation management as described above.

### Clipping Data and Feature Selection

To build a machine-learning-based creeping bentgrass yield prediction model, one of the essential elements is to have a database of the historical clipping records from the field. We collected the creeping bentgrass clippings from the two research greens from 2019 to 2020 (Experiment 1 and 2). The clippings from each research plot were collected approximately every other day between 900 and 1,200 h (weather permitting) by mowing a 1.9 m pass down the center of each plot using a 0.54-m wide walking greens mower (Toro Co., Bloomington, MN, United States). Before the clipping collection, 0.27 m wide alleys were mowed at the top and bottom of each plot perpendicular to the collection pass. This was done to reduce the variability associated with starting and stopping the mower. The effective clipping collection area for each plot was 1 m^2^. The clippings were brushed from the mower bucket into paper bags, which were then placed in an oven set to 50°C for at least 48 h. Sand and other debris were removed from the dried clipping samples using the water method as described by Kreuser et al. (2011). Then, the dry clipping mass was weighed and recorded.

The soil moisture content and normalized difference red edge (NDRE) for each plot were recorded before the clippings were collected. Vegetative indexes such as NDRE and the normalized difference vegetation index (NDVI) started to be widely researched and studies have shown its high correlation with turfgrass quality (Fitz–Rodríguez and Choi, 2002; Bremer et al., 2011). Both NDRE and NDVI rely on different wavelengths of light. NDVI uses near-infrared red light and red light, and can correlate with the vegetative health status at the top of a plant canopy, but may not capture the vegetative health when the canopy has several layers or the leaf area index of the canopy is high. The turfgrass on putting greens is maintained at a very high density. NDRE, which is collected by near-infrared light and a red-edge band (a narrow wavelength between red light and near-infrared red light), may result in a better indication of the vegetative index of the dense turf because the red edge (RE) band penetrates deeper into the turf canopy than the red band of NDVI. Therefore, in this study, we used NDRE to represent the turfgrass health status. The NDRE was collected using a handheld device approximately 1 m above the canopy (Rapid SCAN CS-45, Holland Scientific Inc., Lincoln, NE, United States).

The variables that were used as inputs when predicting the yield included the (1) 3-day average soil moisture content which means the average soil moisture content on the clipping collection event and the one before; (2) average weekly traffic intensity; (3) NDRE; (4) root zone of the two putting greens; (5) cumulative days of turfgrass growth prior to mowing; and (6) daily weather variables which obtained from nearest weather station reported on Weather Underground (an open online real-time weather information repository). These variables included the daily maximum temperature (Tmax), minimum temperature (Tmin), average temperature (Tavg), precipitation (precip), maximum relative humidity (RHmax), minimum relative humidity (RHmin), average relative humidity (RHavg), average wind speed (Windavg), and ET which was from the UW-Extension Ag Weather station. A description of the weather variables utilized is presented in Table 2.

**TABLE 2:** Variables used in the random forest (RF) models.

RF variables	Variables names
Biweekly N rate	N rate (kg/ha/2wk)
Daily NDRE	NDRE
Three-day average soil moisture content	Moist avg (3 days)
Weekly walking traffic	Traffic (round/week)
Accumulative days turf grows	Days grow
Research green soil	Rootzone
Maximum, minimum, and average temperature/relative humidity on the clipping collection day	Tmax; RHmax Tmin; RHmin Tavg; RHmin
Maximum, minimum, and average temperature/relative humidity of x (1,2,3,4,5,6) days before the clipping collection day	Tmax (pre x days) Tmin (pre x days) Tavg (pre x days) RHmax (pre x days) RHmin (pre x days) RHavg (pre x days)
Accumulative maximum, minimum, and average temperature/relative humidity of x (2,3,4,5,6,7) days	Tmax (x days accu) Tmin (x days accu) Tavg (x days accu) RHmax (x days accu) RHmin (x days accu) RHavg (x days accu)
Accumulative precipitation/evapotranspiration of x (2,3,4,5,6,7) days	Precip (x days accu) ET (x days accu)
Accumulative difference between precipitation and evapotranspiration of x (2.3.4.5.6.7) days	Precip-ET (x days accu)
Average wind speed of x (1,2,3,4,5,6) days before the clipping collection day	Wind avg (pre x day)

### Selection and Random Forest Yield Prediction Model

In this study, we tested five machine learning models that have been used for agricultural crop yield prediction, which include RF, gradient boosting model, extreme gradient boosting, decision tree, and support vector regression. After comparing the model performance, we eventually choose RF (Breiman, 2001). Random forest uses a decision tree as a base learner and includes a large set of decision trees, and each tree is independently trained by a random set of variables (listed in Table 2) and corresponding data from the training set. The algorithm first creates a bootstrapped dataset, which requires randomly selected subsets of samples from the original dataset that have the same size, and the number of decision trees would be created based on each subset of data. This step is repeated until the predefined number of trees is reached. In this study, the number of trees was set to 100. The yield prediction was calculated by averaging the predictions of each decision tree. The advantage compared with a single decision tree is that RF can help avoid overfitting (Friedman, 2017).

The “Scikit-learn” RF package from Python (Pedregosa et al., 2011) was used in this study. Considering the availability of the input data from each golf course varies, we built three RF models with different intensities of data complexity and number of features. Three models were created for predicting the creeping bentgrass clipping production, which included the (1) complete RF model, which includes all variables (Table 2) input, (2) simplified RF model, which included all variables except NDRE and 3-day average soil moisture water content, and (3) weather-only RF model, which contained all (and only) weather variables.

**FIGURE 1:**
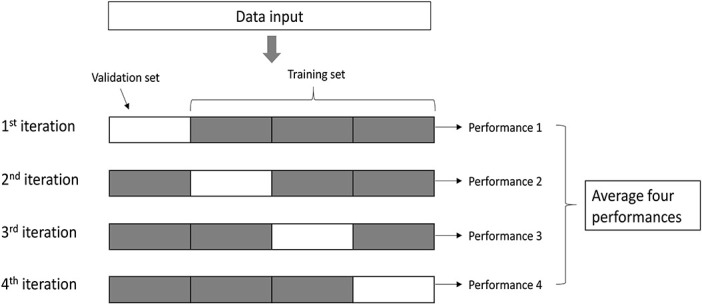
Flow chart of the four-fold cross-validation.

We used the clipping yields collected from 2019 and 2020 (Experiment 1 and 2) to train and validate the model (*n* = 2,190) and explored the predictors including daily weather variables, management practices (soil water content, historical N application rate, and walking traffic), vegetative index data (NDRE), and other parameters that could potentially affect the physiology of the turfgrass (i.e., turfgrass mowing frequency). To validate the model, we used 90% of the total data (*n* = 1,897) to train the model during the training process and the remaining 10% of data (*n* = 293) was used to evaluate the model performance. We adopted four-fold cross-validation to test the performance of the RF model (Figure 1). The dataset was divided into four subsets, and each time one of the four subsets was used as a validation set and the remaining three subsets were used as the training set. This way, every subset was used as a validation set once and as a training set three times.

The hierarchy of the important variables was also determined based on the “scikit-learn” package, which was expressed as a feature importance score. Features with higher scores are important for model accuracy. The coefficient of determination (R^2^) and root mean square error (RMSE) were statistical parameters used for evaluating the accuracy of the models by comparing the predicted values and actual values. To understand the result of the machine learning models, partial dependence plots (PDPs) were used. The PDPs explain how each important variable affects the yield predictions by showing how the target variable partially depends on one or two input variables (Friedman, 2001); therefore, it also can help to visualize and understand whether the relationship between a target and a feature is linear, monotonic, or more complex.

### Golf Course Data for Validating the Model

To test whether the models developed from the research greens data were useful for predicting the yield on the bentgrass putting greens from a different location, we gathered historical clipping records from a golf course in Minneapolis, MN, United States. The putting greens were sand-based soil and constructed according to the recommendations of USGA (similar to our research greens). The clipping yield data were provided as fresh clipping volume instead of dried-and-cleaned clipping mass. However, we were able to convert the fresh clipping volume to dried clipping mass *via* a linear relationship between the two (Supplementary Figure 1). Measuring the fresh clipping volume is less time and labor-intensive than dried clipping mass. The turfgrass manager from the golf course provided the historical N application records. The weather data from the golf course were obtained from Weather Underground. The golf course used plant growth regulators occasionally, so the clipping data were categorized depending on the usage of growth regulators at the time of collection.

## Results

### Model Performance on the Research Greens

Table 3 lists the performance of the five machine-learning models on the turfgrass clipping production. The RF model (*R*^2^ = 0.64) outperformed the single regression models, which included the decision tree (*R*^2^ = 0.36) and support vector regression (*R*^2^ = −0.15), and the boosting ensemble models, which included the gradient boosting model (*R*^2^ = 0.43) and extreme gradient boosting (*R*^2^ = 0.57). Therefore, in the following analysis and discussion section, we deeply investigated the RF model.

**TABLE 3:** Comparison of the performance of five machine learning models for training data set (*n* = 1897).

Machine learning method	R^2^ with SD	RMSE with SD
Random forest (RF)	0.64 (0.08)	0.339 (0.06)
Extreme gradient boosting	0.57 (0.15)	0.366 (0.07)
Gradient boosting model	0.43 (0.13)	0.422 (0.07)
Decision tree	0.36 (0.17)	0.450 (0.09)
Support vector regression	−0.15 (0.15)	0.604 (0.08)

*Full variable inputs were used when developing models.*

**FIGURE 2:**
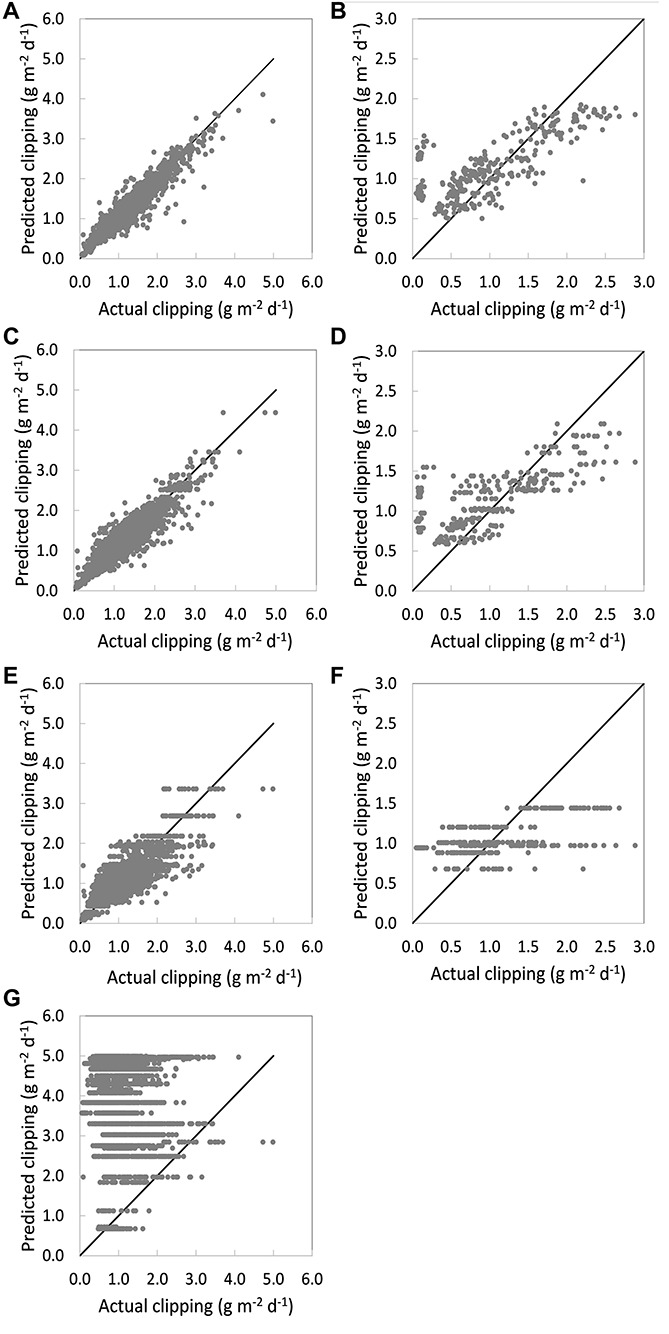
Scatter plot of the model performances with **(A)** complete random forest (RF) model with all variables inputs on the training dataset; **(B)** complete RF model with all variables inputs on the validation dataset; **(C)** simplified RF model with the historical nitrogen (N) rate record, traffic intensity, and weather data on the training dataset; **(D)** simplified RF model with the historical N rate record, traffic intensity, and weather data on the validation dataset; **(E)** simplified RF model with only weather data input on the training dataset; **(F)** simplified RF model with only the weather data input on the validation dataset; **(G)** PACE Turf GP model.

Table 4 lists the three RF model performances on the training dataset (*n* = 1,897) collected from 2019 and 2020 at the University of Wisconsin research station, as well as the PACE Turf GP model performance. During the study period, the daily clipping yield spanned two orders of magnitude (0.09 to 4.1 g m^–2^d^–1^, with 95% of clipping at the range from 0.4 to 3 g m^–2^ d^–1^). The complete RF model that included the entire suite of variables (listed in Table 2) had the best performance (columns 3 and 4 in Table 4 and Figure 2A). Regressed against the actual clipping yield, it had an average R^2^ of 0.64 with an standard deviation (SD) of 0.08 and had the lowest RMSE values compared with the other models created. The simplified RF model was similar to the previously described complete RF model but with no proximal sensing data (NDRE) or soil moisture content input. For the simplified model, the average R^2^ was 0.57 with an SD of 0.09 (Table 4 and Figure 2C). The weather-only RF model only contained weekly weather data inputs and had an average R^2^ of 0.46 with an SD of 0.20 (Table 4 and Figure 2E). The model accuracy decreased with fewer variable inputs.

**TABLE 4:** Model performance on the training and validation datasets of the complete RF model, simplified RF model, and weather-only RF model.

	Variables input	Training RMSE	Training R^2^ with SD	Evaluation RMSE	Evaluation R^2^
Complete RF model	N fertilization Traffic intensity Categorized root zone Weather Days grow NDRE ^*a*^ Soil moisture content	0.339 (0.06)	0.64 (0.08)	0.489	0.47

Simplified RF model	N fertilization Traffic intensity Categorized root zone Weather Days grow	0.367 (0.06)	0.57 (0.09)	0.515	0.42

Weather-only RF model	Weather Days grow	0.406 (0.09)	0.46 (0.20)	0.567	0.30

PACE Turf GP model	Temperature	N/A	0.01	N/A	N/A

*^*a*^NDRE, normalized difference red edge.*

Table 4 also presented the performance of the RF model on the validation datasets (*n* = 293) where the data spanned from 0.10 to 2.89 g m^–2^d^–1^ (columns 5 and 6). The data were collected from 2019 and 2020 at the University of Wisconsin research station. Overall, the complete RF model had the highest coefficient of determination (0.42) and lowest RMSE of 0.49 (Figure 2B). As the number of input variables decreased in the simplified RF model and weather-only RF model, the coefficients of determination also decreased to 0.42 and 0.27, respectively (Figures 2D,F). We compared our statistical models to the PACE Turf GP model that uses the daily average temperature only (Figure 2G). The PACE Turf GP model had the lowest accuracy (*R*^2^ = 0.01), which was unsurprising because that model only uses a single variable (temperature) to predict the clipping yield.

**FIGURE 3:**
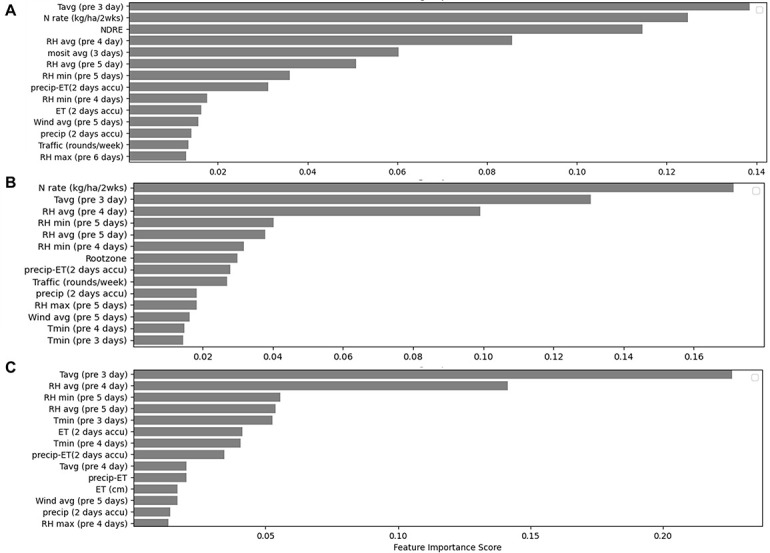
Top important variables for the **(A)** complete RF model; **(B)** simplified RF model with N rates, traffic intensities, and weather inputs; **(C)** weather-only RF model with all the weather variable inputs.

The RF algorithm identifies the relative influence of the factors that affect creeping bentgrass clipping production. In the complete RF model, the top five variables were found to be the (1) average daily air temperature 3 days prior to clipping collection; (2) N fertilizer rate; (3) NDRE; (4) average relative humidity 4 days prior to clipping collection; and (5) 3-day average soil moisture content (Figure 3A). Among the three management practices we investigated in this study (soil moisture, N fertilizer rate, and walking traffic), both the N rate and soil moisture content were found to be more important than traffic in terms of influencing the creeping bentgrass clipping yield. This is aligned with our previously reported findings that when the traffic intensity was maintained at a realistic intensity (0 to 1,800 rounds/week), the effect on the creeping bentgrass growth was small (Zhou and Soldat, accepted), but its influence was much more important than other weather variables like wind speed. In the simplified RF model, besides N fertilizer rate, temperature, and relative humidity, the root zone was another important variable (Figure 3B). Briefly, although the golf course greens were sand-based soil and constructed based on USGA recommendations which had very similar soil texture and soil organic N, soil characteristics such as N mineralization rates could be very different among root zones, and those could potentially result in different plant-available N which would affect the clipping yield. Finally, the key weather variables in the weather-only RF model agreed with the weather variables found to be important in the complete RF model and simplified RF model. Generally, relative humidity and temperature which were observed a few days prior to the collection were the most important weather variables (Figure 3C), demonstrating that weather has a delayed effect on creeping bentgrass clipping yield.

**FIGURE 4:**
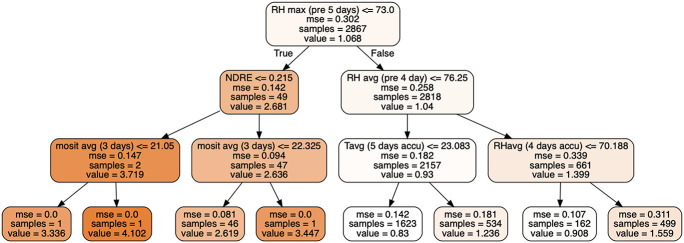
A decision tree with three depths. The nodes with dark colors have a higher estimated clipping yield.

A decision tree was created to visualize how the most important factors were used to predict the creeping bentgrass yield (Figure 4). The first node was split based on one of the most important variables, maximum relative humidity, 5 days prior to the clipping collection event (list as *RH max (pre 5 days)* in Figure 4) with a threshold of 73%. The average clipping yield predictions were 2.68 and 1.04 g m^–2^ d^–1^ as the maximum relative humidity was below or above 73%. The decision tree was further divided based on NDRE, RH avg (listed as *pre 4 day* in Figure 4), most avg (listed as *3 days* in Figure 4), Tavg (listed as *5 days accu* in Figure 4), and Rhavg (list as *4 days accu* in Figure 4). Overall, this single decision tree presents an example of how the RF algorithm would use the decision tree as a base learner to predict creeping bentgrass clipping yield.

**FIGURE 5:**
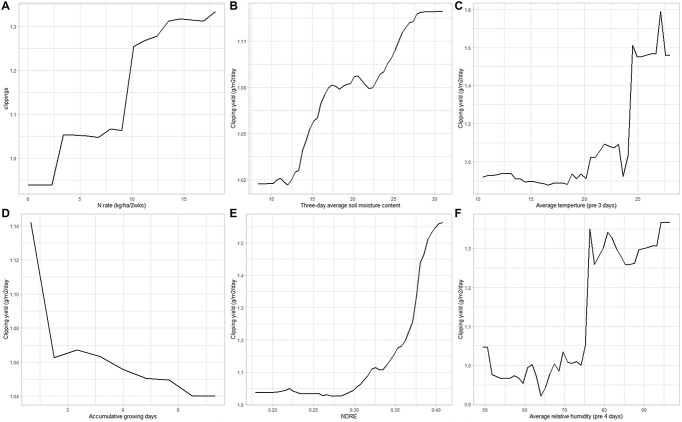
The partial dependence of creeping bentgrass clipping production on the N application rate **(A)**; average 3-day soil moisture content **(B)**; the average temperature on the previous 3 days of clipping collection event **(C)**; accumulative days that turf grew between mowing **(D)**; normalized difference red edge (NDRE) **(E)**; average relative humidity on the previous 4 days of clipping collection event **(F)**.

PDPs were created for the top five most important variables among three RF models (complete RF model, simplified RF model, and weather-only RF model) to understand the relationship between the most important features and creeping bentgrass clipping production. Moreover, in this study, the mowing frequency (or cumulative days that turfgrass grown between two mowing events) varied and the cumulative growing days include 1, 2, 3, 4, and 5 days. Therefore, we included the cumulative turfgrass growing days and expected to find a relationship between mowing frequency and turf growth. Nitrogen application rate and soil moisture content were positively correlated with creeping bentgrass clipping production (Figures 5A,B). When the soil moisture tripled from 10 to 30%, the clipping production only increased about 10% (from 1.02 to 1.12 g m^–2^d^–1^). It agreed with our previous result that the influence of soil moisture content on the growth rate of creeping bentgrass was discernable but small (Zhou and Soldat, accepted). The NDRE of the plots spanned from 0.14 to 0.41, and it was also positively correlated with the creeping bentgrass growth (Figure 5E) especially when the NDRE spanned from 0.28 to 0.41. We found little increase in the clipping yield when the NDRE was at the range of 0.14 to 0.28. The turf mowed more frequently had a lower clipping yield than the turf mowed less frequently (Figure 5D). This could be explained by one of the assumptions of the mechanism of plant defense that plants would grow fast to recover from the mowing damage (Stamp, 2003).

The two important weather variables (temperature and relative humidity) displayed a more complex relationship with bentgrass yield. Creeping bentgrass growth peaked when the temperature on the third day prior to clipping collection was around 25–27°C (Figure 5C), significantly higher than the 20°C assumed by the PACE Turf GP model for cool-season grasses. As the temperature increased from 10 to 23°C, the PDPs showed that there was a small increase in the clipping production, but a larger increase in the clipping yield when the temperature increased from 23 to 25°C. Similarly, there was a steep increase in clipping production when the relative humidity was above 75% (Figure 5F). When the relative humidity was below or above 75%, we found very little impact on the clipping yield.

**FIGURE 6:**
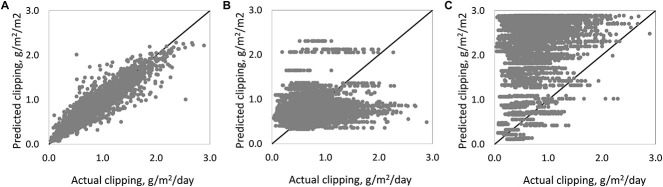
Scatter plot with **(A)** random forest (RF) model performance from the MN golf course that was built with on-site clipping data; **(B)** RF model built with clipping data collected from Madison, Wisconsin, USA; **(C)** PACE Turf GP model.

RF models that were built based on the data collected from the University of Wisconsin-Madison research site in 2019 and 2020 were used to predict the clipping yield on bentgrass putting greens from a golf course located in Minnesota, USA. Since the golf course had accesss to only historical N fertilization rate and weather, we used the simplified RF model to make predictions. When converting fresh clipping volume to dried clipping mass, a conversion of 0.57 was used (Supplementary Figure 1). The clipping yield overall was similar to the ranges we found on our plots and also spanned two orders of magnitude from 0.03 to 2.89 g m^–2^ d^–1^, (*n* = 2190, with 95% of clipping at the range from 0.3 to 2 g m^–2^ d^–1^). The simplified RF model built based on the data collected from the Wisconsin research putting greens performed poorly with an *R*^2^ of 0.03 (Figure 6B). The PACE Turf GP model also had relatively low prediction accuracy (*R*^2^ = 0.05) on the turfgrass clipping production (Figure 6C). However, a customized RF model based on the Minnesota data was constructed using the clipping volume data collected from the golf course from Minnesota, USA. This model predicted clipping yield well with an *R*^2^ of 0.74 (Figure 6A). While we failed to create a universal statistical bentgrass yield prediction model, we have demonstrated that it is possible to build accurate, customized growth models with local clipping data and readily available input variables like weather data and N fertilization rate.

## Discussion

Accurate turfgrass yield prediction would enable early and accurate decision-making and allow managers to more sustainably manage fertilizer resources. This study used clipping yield data from field experiments, and the results have shown that the interactions among environmental factors, various management practices, and clipping yield were rather complex and had non-linear relations. Three RF models were created based on the experimental results. Our results showed that the complete RF model with inputs of weather variables, NDRE, and management practices (N fertilization rate, mowing frequency, traffic intensity, and irrigation plan) could provide a relatively high accuracy turfgrass clipping yield prediction model.

In this study, we found that NDRE values were a critical parameter for creeping bentgrass yield prediction, and it represented the canopy information related to the density and vigor of turfgrass. The RF model also listed NDRE as one of the most important features. If mowers can be equipped with proximal sensors that can collect NDRE and other vegetation indexes, it will become easier for managers to improve their own statistical models that are predictive of yield to more effectively manage N fertilization.

When evaluating the weather variables, temperature and relative humidity were all highly associated with the turfgrass growth rate or clipping production. The correlation between these weather variables and the creeping bentgrass clippings was rather complex and weak compared with other variables it explained the failure of the existing growth rate prediction model. Solar radiation, an important factor influencing plant growth, was not included in this study because these data are not readily available to most turfgrass managers. Overall, the weather had a delayed effect on creeping bentgrass clipping yield, demonstrating that weather factors prior to the clipping collection usually have a greater impact on growth compared with the impact of weather factors collected on the day of or the day prior to clipping collection.

The RF model has been used to predict annual or seasonal agricultural crop yield with high accuracy (Everingham et al., 2016; Maya Gopal and Bhargavi, 2019; Zhang et al., 2019). This study also verified that the RF model was able to provide high prediction accuracy compared with the other four commonly used machine learning models that include the decision tree, gradient boosting model, extreme gradient boosting, and supper vector regression. The RF model succeeded in the prediction of the short-term turfgrass clipping yield for both the plot-scale clipping data and golf course green-scale clipping data. The RF model is also computationally fast (Ziegler and König, 2014) and simple to operate. Moreover, the simplified RF model was also tested to have a relatively high prediction accuracy. This simplified model could provide valuable clipping yield prediction for golf courses that do not have access to some of the more intensive variables tested in this study like NDRE and soil moisture content.

With the increasing attention on resource use efficiency, more quantitative methods for guiding N application are required. Similar to agricultural crop management, yield prediction is also essential for the decision-making and N management of turfgrass managers. This study is the first to predict turfgrass clipping yield with machine-learning approaches and found that the RF algorithm was the most useful. For the future precision of N management on golf courses, it is evident that future improvements such as the incorporation of sensors could be beneficial. Although our goal of constructing a universal bentgrass yield prediction model was not achieved, individual golf courses could build customized yield prediction models with sound accuracy by using their own yield measurements. Therefore, for future research, there is a need to investigate the feasibility of using machine-learning techniques, specifically the RF model, in guiding N application decisions compared with the existing N application strategies under field conditions. Additionally, a precision fertilization plan requires the understanding of plant nutrient needs, their response to different nutrient applications, and the supply of N from indigenous sources (Dobermann et al., 2003). Future studies should also seek to quantify soil mineralized N, which will provide a more complete understanding of soil-turfgrass-environment interactions and lead to more efficient use of fertilizer inputs.

## Conclusion

The machine-learning models were effective for turfgrass yield prediction. As the first study to develop machine-learning models to predict turfgrass yield, we concluded that the RF model resulted in the greatest accuracy to predict creeping bentgrass clipping yield. Three RF models with different intensities of data complexity were presented. The results demonstrated that the model with the greatest number of inputs had the greatest accuracy and the models with a reduced number of inputs, particularly those missing soil and vegetation sensed data, had lower yield prediction accuracy. However, all three RF models outperformed the current growth prediction model which used only temperature to estimate turfgrass yield. These findings suggest that golf course managers will be able to better estimate turfgrass growth, even with limited access to input variables. Additionally, our study showed that weather had a delayed effect on turfgrass growth, and the use of 1-day weather data would not result in the best yield prediction as to the inclusion of multi-day weather data. Using an RF algorithm to build an accurate yield prediction model narrows the knowledge gap for accurate N application guidance to achieve site-specific N management. The RF model was successful in providing an accurate estimation of the clipping yield at a fine-scale which may assist turfgrass managers to more effectively allocate resources and modify management practices site-specifically.

## Data Availability Statement

The original contributions presented in the study are included in the article/Supplementary Material, further inquiries can be directed to the corresponding author.

## Author Contributions

QZ and DS designed the study and conducted the field experiments. QZ performed the data analysis, built the prediction model, and wrote the manuscript. DS provided critical insights, edited, and revised the manuscript. All authors reviewed the manuscript and agreed with the submission.

## Conflict of Interest

The authors declare that the research was conducted in the absence of any commercial or financial relationships that could be construed as a potential conflict of interest.

## Publisher’s Note

All claims expressed in this article are solely those of the authors and do not necessarily represent those of their affiliated organizations, or those of the publisher, the editors and the reviewers. Any product that may be evaluated in this article, or claim that may be made by its manufacturer, is not guaranteed or endorsed by the publisher.
