# Corrigendum: Creeping Bentgrass Yield Prediction With Machine Learning Models

**DOI:** 10.3389/fpls.2021.829508

**Published:** 2022-01-21

**Authors:** Qiyu Zhou, Douglas J. Soldat

**Affiliations:** Department of Soil Science, University of Wisconsin–Madison, Madison, WI, United States

**Keywords:** turfgrass, nitrogen management, yield prediction, machine learning, random forest

In the original article, there was a mistake in the legend for Figure 6 as published. The legend of Figure 6 did not match the figure, and panels (A), (B), and (C) were written in the wrong order. The correct legend appears below.

“Scatter plot with **(A)** random forest (RF) model performance from the MN golf course that was built with on-site clipping data; **(B)** RF model built with clipping data collected from Madison, Wisconsin, USA; **(C)** PACE Turf GP model.”

In the original article, there was an error. As the legend of Figure 6 was incorrect, the corresponding text was also incorrect.

A correction has been made to Results, “*Model Performance on the Research Greens”*, paragraph 8:

“RF models that were built based on the data collected from the University of Wisconsin-Madison research site in 2019 and 2020 were used to predict the clipping yield on bentgrass putting greens from a golf course located in Minnesota, USA. Since the golf course had accesss to only historical N fertilization rate and weather, we used the simplified RF model to make predictions. When converting fresh clipping volume to dried clipping mass, a conversion of 0.57 was used (Supplementary Figure 1). The clipping yield overall was similar to the ranges we found on our plots and also spanned two orders of magnitude from 0.03 to 2.89 g m^−2^ d^−1^, (*n* = 2190, with 95% of clipping at the range from 0.3 to 2 g m^−2^ d^−1^). The simplified RF model built based on the data collected from the Wisconsin research putting greens performed poorly with an *R*^2^ of 0.03 (Figure 6B). The PACE Turf GP model also had relatively low prediction accuracy (*R*^2^ =0.05) on the turfgrass clipping production (Figure 6C). However, a customized RF model based on the Minnesota data was constructed using the clipping volume data collected from the golf course from Minnesota, USA. This model predicted clipping yield well with an *R*^2^ of 0.74 (Figure 6A). While we failed to create a universal statistical bentgrass yield prediction model, we have demonstrated that it is possible to build accurate, customized growth models with local clipping data and readily available input variables like weather data and N fertilization rate.”

The authors apologize for this error and state that this does not change the scientific conclusions of the article in any way. The original article has been updated.

## Publisher's Note

All claims expressed in this article are solely those of the authors and do not necessarily represent those of their affiliated organizations, or those of the publisher, the editors and the reviewers. Any product that may be evaluated in this article, or claim that may be made by its manufacturer, is not guaranteed or endorsed by the publisher.

