# Supplementary material for: Creeping Bentgrass Yield Prediction With Machine Learning Models
Source: Front Plant Sci. 2021 Nov 4;12:749854. doi: 10.3389/fpls.2021.749854 (PMC8600360; doi:10.3389/fpls.2021.749854)
Supplement: Supplementary file 1 [file Data_Sheet_1.docx]

# **Supplementary Material**


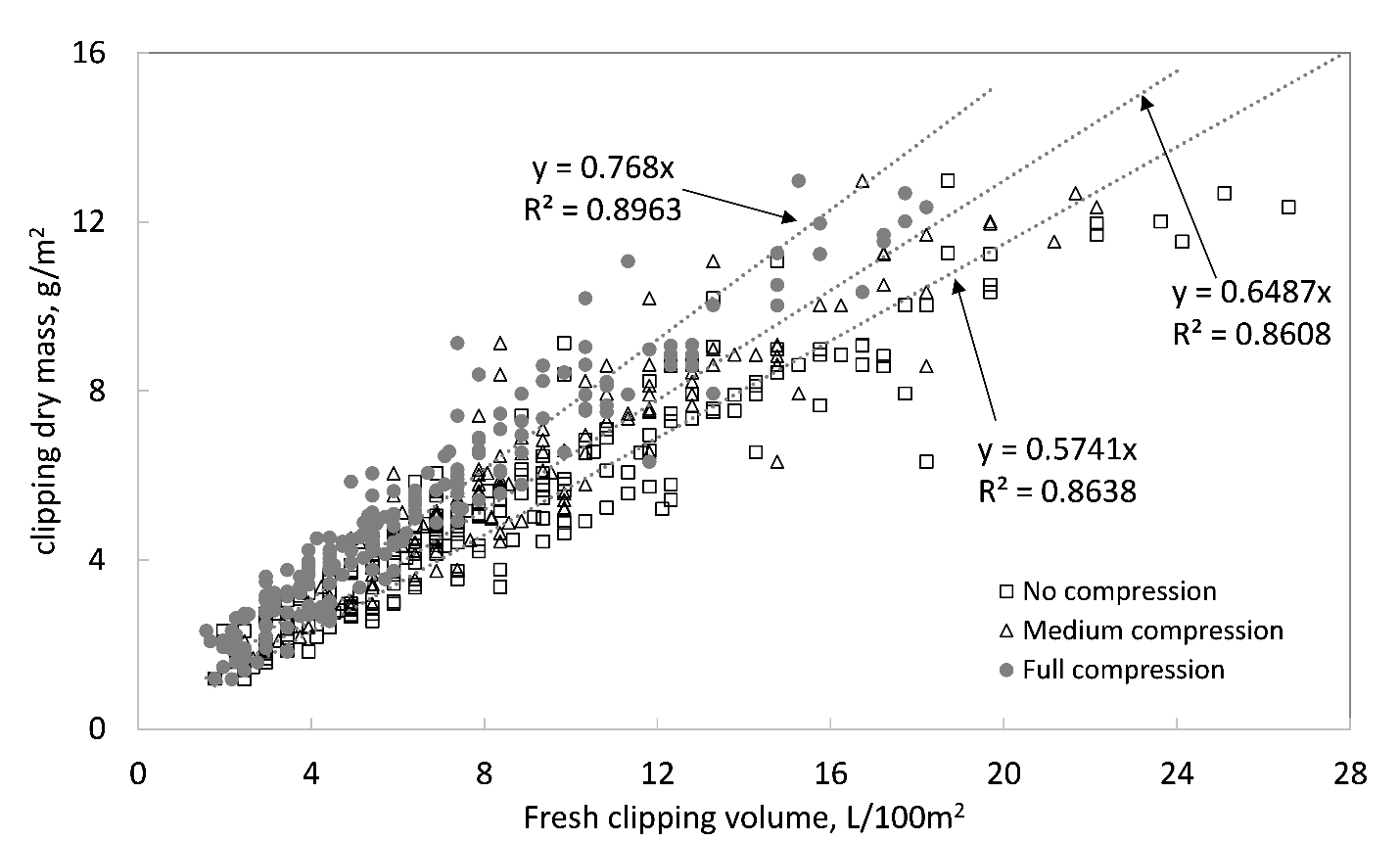


Fig. S1. Conversion between creeping bentgrass clipping dry mass and fresh clipping volume. Open square symbols represent the clipping was transferred from mower clipping bucket to container with scale receiving no compression (R^2^ = 0.86); open triangle symbols represent the clipping was tapped twice and received some compression from gravity (R^2^ = 0.86); filled circle symbols represent the clipping was fully compressed by gravity but did not receive force from pressing on the top (R^2^ = 0.90).
